# A National Observational Study From 2010 to 2021 of the Trends in the Timing of Hip Surgery in Children With Cerebral Palsy: Is Surgery Being Performed Earlier?

**DOI:** 10.7759/cureus.57536

**Published:** 2024-04-03

**Authors:** Anthony K Chiu, Sarah Dance, Samantha L Ferraro, Alana O'Mara, Savyasachi C Thakkar, Sean Tabaie

**Affiliations:** 1 Orthopaedic Surgery, George Washington University School of Medicine and Health Sciences, Washington DC, USA; 2 Orthopaedic Surgery, Children’s National Hospital, Washington DC, USA; 3 Orthopaedic Surgery, Johns Hopkins Health System, Baltimore, USA; 4 Orthopaedic Surgery, Children's National Hospital, Washington DC, USA

**Keywords:** hip displacement, surgical timing, hip surveillance, hip dislocation, cerebral palsy (cp)

## Abstract

Background

Hip instability is a concern in pediatric cerebral palsy (CP) patients, with approximately one-third developing hip displacement. This may lead to pain, functional limitations, and decreased quality of life. Due to the progressive nature of hip displacement in CP, earlier surgical interventions may be beneficial. However, any shifts in practice to earlier surgical intervention, on a national scale, is not well described. The purpose of this study was to determine the recent trends in the surgical timing of hip interventions in children with CP.

Methods

A retrospective study was conducted using the PearlDiver Mariner all-payer claims database (PearlDiver Technologies, Colorado Springs, Colorado, United States). CP patients aged 10 years and younger were identified between 2010 and 2021. Hip surgeries including open reduction, adductor tenotomy, and pelvic osteotomy were identified. Patients were stratified by their age on the date of surgery and the year of the procedure. Linear regression analysis was conducted for temporal trends. Further, the compounded annual growth rate (CAGR) was calculated.

Results

A total of 309,677 CP patients were identified. For those aged one to four years old, the percentage undergoing hip surgery increased from 10.2% in 2010 to 19.4% in 2021. In the five- to 10-year-old age group, the surgery rate peaked at 14.9% in 2016 and steadily declined to 11.5% in 2021. The overall CAGR from 2010 to 2021 was +6.03% for the one- to four-year-old group and +0.88% for the five- to 10-year-old group. Linear regression demonstrated a significant association between year and the percentage of operations for patients ages one to four (R^2^=0.792, p<0.001), but not ages five-10 (R^2^=0.019, p=0.704).

Conclusions

Rates of surgical hip procedures in one- to four-year-old CP patients have been increasing since 2010, whereas the rate in five- to 10-year-old CP patients has been decreasing since 2016. Recently, CP patients may be undergoing hip surgery at younger ages.

## Introduction

In patients with cerebral palsy (CP), spasticity in the muscles around the hip commonly leads to hip displacement. About one-third of children with CP will develop hip displacement and 10-15% of CP patients experience complete hip dislocation [1]. Patients with a dislocated hip often experience severe pain, contractures, and functional limitations, including difficulty sitting, standing, and walking, ultimately requiring surgery [2]. Early screening of hip displacement in children with CP is an effective tool to allow timely intervention when deemed necessary [3,4]. The pressing need to improve hip displacement screening in the United States CP population has in the past decade led to efforts to establish a nationwide surveillance program like the ones that exist in Europe, Australia, and parts of Canada [5].

The successes of CP hip surveillance programs in several other countries have been reported. Sweden’s CP Follow-Up (CPUP) Program includes about 95% of Swedish children with CP who were born in 2000 or later and involves regular radiographic examinations at frequencies determined by age and Gross Motor Function Classification System (GMFCS) level [6,7]. Within 10 years of CPUP implementation, the incidence of hip dislocations among children with CP in Sweden dropped from 9.0% to 0.4%, coinciding with a 1.5% increase in soft tissue surgery and a minimal decrease in reconstructive and palliative surgeries [3,8]. Similar surveillance programs have been initiated in Norway and Scotland [9,10]. These programs are carried out by physical therapists at routine visits and thus require very few additional resources to implement.

While the United States does not have a universal hip screening program for children with CP, pediatric orthopedic surgeons across the country are increasingly invested in early detection and treatment of hip displacement. After surveying members of the Pediatric Orthopedic Society of North America (POSNA), Shore et al. found that 93% of respondents were in favor of a national surveillance program for hip dislocation prevention in CP patients, and 93% would abide by this type of program if implemented [11].

While the prevalence and protocols of hip surveillance in the CP population across the United States are difficult to quantify due to the segmented healthcare system, we can quantify treatment trends. If screening for hip displacement occurs earlier, more thoroughly, or more often in patients with CP, we would expect to see earlier operative treatment as well. These procedures generally consist of open reduction, adductor tenotomy, and pelvic osteotomy [12,13]. Therefore, the purpose of this study is to observe national trends in the percentage of at-risk patients undergoing surgical procedures for hip displacement in the CP population, based on defined age groups. We hypothesize that there has been a shift toward patients undergoing operative hip displacement treatment at a younger age over the past decade.

## Materials and methods

Study design

This was a retrospective observational study with national insurance claims data. Trends in the timing of hip surgeries in cerebral palsy patients were demonstrated through observations of hip surgery stratified by record year and patient age at the time of the procedure. We report surgical interventions within one-to-two-year age strata for the years 2010 to 2021, reported as percentages of the at-risk population.

Data source

This study used a large database to observe national trends over the years 2010 to 2021. We collected data from the PearlDiver Mariner Patient Claims Database (PearlDiver Technologies, Colorado Springs, Colorado, United States), which is a dataset composed of over 157 million patients. At the time of the data collection, the Mariner dataset contained medical and prescription information from January 2010 to October 2021. Multiple payer types are represented, including private insurance, Medicare, Medicaid, and cash. Unique patient identifiers are utilized in order to minimize loss to follow-up from changes in insurance status, thus enabling longitudinal tracking. The data is sourced from provider networks throughout the United States, including representation of all 50 states. 

Patient selection

Patients were identified via the International Classification of Diseases (ICD) 9th and 10th Revision codes and Current Procedural Terminology (CPT) codes. Inclusion criteria consisted of patients with cerebral palsy (ICD-9 343.0-343.9 and ICD-10 G800-G809) who underwent surgical hip interventions including adductor tenotomy, open reduction of hip dislocation, and osteotomy of the femur or pelvis. These procedures were identified via CPT codes provided in Table 1. Pediatric patients aged 10 years or younger were included, as it has been shown that the majority of hip dislocations occur in patients 10 years old or younger [14]. Only patients who underwent a first instance of one of the procedures were included. Therefore, the study population represents patients who received primary hip surgeries.

**Table 1:** Codes used for patient identification ICD: International Classification of Diseases; CPT: Current Procedural Terminology [15]

Group	Codes used
Cerebral palsy	ICD-9-D-343.0:ICD-9-D-343.9, ICD-10-D-G800:ICD-10-D-G809
Open reduction of hip dislocation	CPT-27258, CPT-27259
Adductor tenotomy	CPT-27001
Hip osteotomy	CPT-27165, CPT-27146, CPT-27147, CPT-27151, CPT-27156

Outcomes of interest

The primary outcome of interest in this study was the recent trend in the timing of hip surgery in cerebral palsy patients, defined as the relative change in prevalence by age group from 2010 to 2021 as measured by the compounded annual growth rate (CAGR). Secondary outcomes of interest were the relative changes in hip surgery prevalence stratified by procedure type (open reduction, adductor tenotomy, and osteotomy).

Statistical analysis

The yearly observed prevalence was reported as the percentage of patients who underwent a surgical hip procedure divided by the total number of patients at risk, multiplied by one hundred percent. The CAGR was calculated to summarize the annualized change over time in the percentage of hip surgery observations. The CAGR is a useful summary statistic in trends analysis, allowing for the translation of overall trends to a standardized incremental change. The CAGR is calculated as CAGR = (Y2/Y1)^[1/(Y2-Y1)] - 1, where Y2 is the final year observed and Y1 is the initial year observed. Linear regression analysis was used to determine the univariate relationship between the time (year of procedure) and the observed prevalence of primary hip surgery. Significance was set at an a priori alpha of 0.05.

## Results

Patient population

A total of 309,667 patients aged 10 years or younger were identified who had a diagnosis of cerebral palsy. A total of 131,010 patients were younger than five years of age, and 178,657 were aged five to 10 years old. The sample size was well distributed among years 2010-2021 (Table 2).

**Table 2:** Cerebral palsy patients identified by age and year

		Patient age in years									
Year	Total	<1	1	2	3	4	5-6	7-8	9-10	<5	5 to 10
Total	309,667	3,029	17,541	30,799	38,139	41,502	59,606	59,924	59,127	131,010	178,657
2010	31,303	420	1,990	3,026	3,354	3,242	6,512	6,321	6,438	12,032	19,271
2011	33,517	355	1,858	3,110	3,623	3,722	7,274	6,909	6,666	12,668	20,849
2012	35,049	391	1,919	3,021	3,690	3,957	7,873	7,265	6,933	12,978	22,071
2013	37,283	370	1,907	3,111	3,745	4,022	8,715	8,013	7,400	13,155	24,128
2014	37,660	317	1,749	3,008	3,622	4,055	8,744	8,398	7,767	12,751	24,909
2015	36,705	280	1,654	2,873	3,478	3,773	8,275	8,589	7,783	12,058	24,647
2016	36,347	235	1,449	2,693	3,406	3,687	8,059	8,579	8,239	11,470	24,877
2017	35,061	191	1,290	2,474	3,191	3,589	7,767	8,124	8,435	10,735	24,326
2018	32,523	166	1,253	2,333	2,891	3,209	7,145	7,529	7,997	9,852	22,671
2019	31,918	135	1,083	2,215	2,916	3,137	6,996	7,628	7,808	9,486	22,432
2020	27,229	131	776	1,644	2,350	2,824	6,082	6,640	6,782	7,725	19,504
2021	24,153	38	613	1,291	1,873	2,285	5,658	6,038	6,357	6,100	18,053

Trends in surgical timing

**Figure 1:**
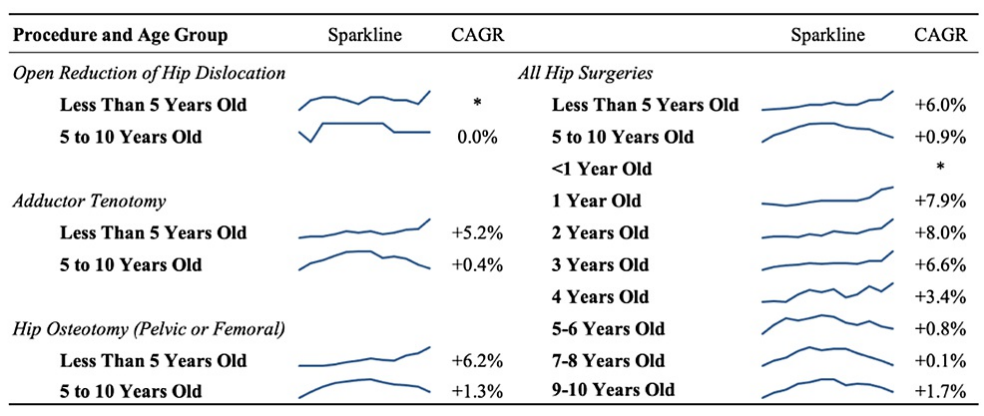
Sparklines of trends in hip surgery for cerebral palsy patients by age from 2010 to 2021 *Unable to be calculated due to a starting value of 0. CAGR: compounded annual growth rate

**Figure 2:**
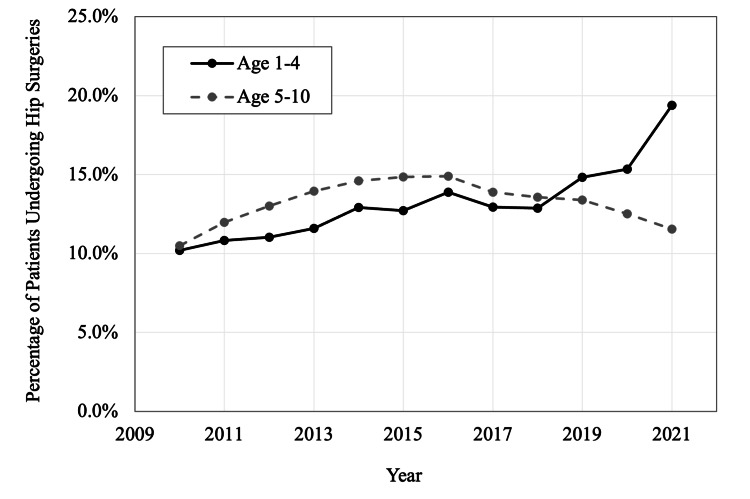
Yearly percentage of hip surgeries in cerebral palsy patients, by age group, in the United States from 2010 to 2021

Overall, an average of 13.2% of all patients included between 2010 and 2021 underwent a primary hip procedure. The ages with the greatest annualized growth in hip surgery volume were patients three years old (CAGR: +6.6%), two years old (CAGR: +8.0%), and one year old (CAGR: +6.6%) (Table 3, Figure 1). When grouped into two evenly divided age groups (a younger group of patients aged less than five years old, and an older group of patients aged five to 10 years), there was a significant increase in hip surgeries over time in the younger group (CAGR: +6.0%, R2=0.792, p<0.001). For patients in the younger group, the percentage of patients undergoing primary hip surgery increased from 10.2% in 2010 to 19.4% in 2021. For patients in the older group, there was no significant overall trend in hip surgery volume between the years 2010 and 2021 (CAGR: +0.9%, R2=0.019, p=0.704) (Table 4). However, the percentage of patients in the older age group undergoing primary hip surgery decreased steadily from 14.9% in 2016 to 11.5% in 2021 (Figure 2).

**Table 3:** Yearly percent prevalence of hip surgery in cerebral palsy patients by age in the United States from 2010 to 2021 Hyphens represent values unable to be calculated due to low patient counts limiting database reporting. *Unable to be calculated due to a starting value of 0 CAGR: compounded annual growth rate

Procedure and age group	Average	2010	2011	2012	2013	2014	2015	2016	2017	2018	2019	2020	2021	CAGR
Open reduction of hip dislocation (%)														
<5	0.3	0	0.3	0.4	0.4	0.3	0.2	0.4	0.4	0.3	0.3	0.2	0.6	*
5 to 10	0.4	0.4	0.3	0.5	0.5	0.5	0.5	0.5	0.5	0.4	0.4	0.4	0.4	0.0%
Adductor tenotomy (%)														
<5	6.3	5.1	5.4	5.5	5.8	6.4	6.1	6.4	5.9	6.1	6.8	6.9	8.9	+5.2%
5 to 10	6.1	4.9	5.6	5.9	6.4	6.8	6.9	6.9	6.2	6.3	6.1	5.5	5.1	+0.4%
Hip osteotomy (pelvic or femoral, %)														
<5	6.6	5.1	5.1	5.2	5.5	6.1	6.4	7.1	6.7	6.5	7.7	8.3	9.9	+6.2%
5 to 10	6.7	5.2	6	6.6	7.1	7.3	7.5	7.6	7.2	6.9	6.8	6.6	6.0	+1.3%
All hip surgeries (%)														
<5	13.2	10.2	10.8	11	11.6	12.9	12.7	13.9	12.9	12.9	14.8	15.4	19.4	+6.0%
5 to 10	13.2	10.5	12	13	14	14.6	14.8	14.9	13.9	13.6	13.4	12.5	11.5	+0.9%
<1	5.2	6.7	3.4	8.7	3	3.5	3.9	6	6.3	-	-	-	-	-
1	12.6	9.8	8.9	7.7	8.9	10.5	11.7	12	12.2	12.1	14	20.6	22.7	+7.9%
2	12.6	9.2	10.4	10.3	9.6	11.5	10.9	13.7	12.6	12	14.3	15.4	21.4	+8.0%
3	13.4	9.8	11.3	12.3	12.6	13.3	13.1	13.5	13.4	13	14.4	14.7	19.8	+6.5%
4	14.5	12.1	12.4	12.2	14.3	15.4	14.8	15.6	13.4	14.3	16.5	15.1	17.4	+3.4%
5-6	12.3	10.4	11.9	13.1	12.7	13.1	13.6	13.3	12.3	11.9	12.5	11.7	11.3	+0.8%
7-8	13.5	11	12.2	13	14.6	15.5	14.8	15.1	15	14	13.1	12.3	11.2	+0.1%
9-10	13.9	10.1	11.8	12.9	14.7	15.3	16.2	16.3	14.3	14.7	14.5	13.5	12.1	+1.7%

**Table 4:** Compounded annual growth rate and linear regression analysis of all hip surgeries in cerebral palsy patients aged less than five years old versus cerebral palsy patients aged five to 10 years old *Linear regression. Bolded values represent significance below the threshold of p=0.05. N: number of patients; CAGR: compounded annual growth rate

	Age less than five years			Age five-10 years		
	Total	All hip surgeries		Total	All hip surgeries	
Year	N	N	Percent	N	N	Percent
2010	12,032	1,226	10.2%	19,271	2,022	10.5%
2011	12,668	1,372	10.8%	20,849	2,495	12.0%
2012	12,978	1,431	11.0%	22,071	2,871	13.0%
2013	13,155	1,526	11.6%	24,128	3,366	14.0%
2014	12,751	1,647	12.9%	24,909	3,637	14.6%
2015	12,058	1,533	12.7%	24,647	3,658	14.8%
2016	11,470	1,593	13.9%	24,877	3,705	14.9%
2017	10,735	1,390	12.9%	24,326	3,377	13.9%
2018	9,852	1,269	12.9%	22,671	3,077	13.6%
2019	9,486	1,406	14.8%	22,432	3,003	13.4%
2020	7,725	1,186	15.4%	19,504	2,441	12.5%
2021	6,100	1,183	19.4%	18,053	2,085	11.5%
Mean ± SD			13.2 ± 2.5%			13.2 ± 1.4%
CAGR			+6.0%			+0.9%
R^2^			0.792			0.019
p-value*			<0.001			0.704

Trends by surgical procedure

Among the surgical hip procedures investigated, all procedures trended toward an increase in volume in younger patients after sub-stratification. The rate of open reduction for hip dislocation increased from an observed 0.3% in 2011 to 0.6% in 2021. Of note, no open reductions were identified in the database for 2010 due to sample unavailability, which limited the CAGR analysis for overall trends. The percentage of patients aged five years or younger who underwent adductor tenotomy increased from 5.1% in 2010 to 8.9% in 2021 (CAGR: +5.2%). Patients aged five or younger who underwent pelvic or femoral osteotomy increased from 5.1% in 2010 to 9.9% in 2021 (CAGR: +6.2%). For patients ages five to 10 years old, there were small incremental increases in the percentages of patients who underwent open reduction (CAGR: +0.7%), adductor tenotomy (CAGR: +0.4%), and osteotomy (CAGR: +1.3%) (Table 3, Figure 1).

## Discussion

The results of this study show a nationwide trend that pediatric CP patients are undergoing surgical hip interventions at younger ages. The percentage of surgical hip procedures has been increasing since 2010 in one- to four-year-old CP patients with a CAGR of +6.0%. In contrast, the percentage in five- to 10-year-old CP patients peaked in 2016 and has since been decreasing. While the CAGR from 2010-2021 in this age group was +0.9%, suggesting future growth in the number of CP patients receiving hip surgeries, the decline that occurred in 2016 would likely cause a negative CAGR from 2016-2021.

The increase in surgical hip interventions in younger patients is likely due to a multitude of factors. One factor may include the adoption of hip surveillance programs by the United States practicing physicians. In 2016, 18% of respondents to a Pediatric Orthopaedic Society of North America (POSNA) survey noted that they followed a strict hip surveillance program, most commonly the Australian protocol [14]. The trends seen in this study may provide evidence that hip surveillance programs in the United States have become more popular and are beginning to influence the age of surgical interventions.

The success of hip surveillance programs in other countries, as previously mentioned, has been well documented. As well as improved classification of hip pathology in CP patients, hip surveillance programs have led to earlier diagnosis and reduced risk of hip dislocation, mitigating the risk of dangerous sequelae related to dislocation [4,6,8]. Global hip surveillance programs have shown similar results regarding surgical rates to our own study [4,16,17]. For example, a study comparing the prevalence of hip dislocation in pediatric CP patients in Norway without a hip surveillance program versus in Sweden with a hip surveillance program showed that initial hip surgery was performed at a younger age in the population with the formal surveillance program [16]. While this is congruent with our study, it is important to note that the European study had a much smaller population size of patients that underwent surgical intervention compared to our study, and the mean age of operation in the Swedish group was 5.7 years old, while our youngest age group only went to age four. A larger study also conducted in Sweden including 3,305 children and adolescents similarly demonstrated that those less than 15 years of age more frequently underwent surgical procedures and had lower gross motor function scores, corroborating the early intervention seen in our study [18].

The decreasing trend of surgical hip interventions performed in the older age group since 2016 cannot be clearly explained based on our data, but one could speculate that it is related to the rise of surgeries in the younger patient population. As patients receive surgical hip intervention earlier, there may be less of a need for intervention later. However, this raises questions about the efficacy and safety of surgical hip interventions in a younger patient population. It could be that earlier identification of hip displacement and earlier surgical intervention are better at managing the progression of migration percentage (MP), thereby preventing early hip dislocation and the need for more invasive procedures. There are several studies that have advocated for early soft tissue releases to prevent early hip dislocation and the need for bony reconstructions [19-21].

Other studies have shown that early surgery is associated with a high risk of reoperation. In a Swedish study following pediatric CP patients five years after primary hip surgery, researchers found a high risk of reoperation rate that had a significant correlation to MP and less of a correlation to age [22]. Shore et al. (2015) reviewed factors affecting surgical success following femoral varus derotational osteotomy (VDRO) in children with CP, utilizing multivariate analysis to control for potential confounders. In contrast with other studies, Shore et al. noted that younger age at surgery, increased GMFCS level, and lower annual surgical hip volume were significantly associated with the increased rate of surgical revisions [23]. Because of these conflicting results, more current data is needed to establish the relationship between age and surgical hip intervention success.

While the study findings are consistent with our initial hypothesis, there are several limitations of the present study to address. Given the nature of retrospective observational studies and the use of ICD and CPT codes, the results may include incomplete data or misclassified patients, are subject to confounding, and do not establish causation. Our study also did not evaluate surgical rates based on other patient characteristics, such as GMFCS score, sex, or race. Furthermore, the age groups used in this study were determined prior to data analysis, and adjusting these age groups may alter our results. 

This study establishes that there has been a shift in the timing of surgical hip intervention, but the factors contributing to this shift need further investigation. Given the obscure age groups set in the study, further analysis of the data for different age groups and by different procedures should be pursued. Future prospective research may help to determine if the surge of hip surveillance programs in the United States affects the age at which surgical hip interventions are being performed. Finally, surgical hip interventions in a younger CP population should be evaluated for efficacy and safety given some past studies that have shown an increased risk of hip displacement recurrence at younger ages of intervention, as stated above. Despite the limitations of this study and the need for further research, it is important to note that this is one of the first studies, to our knowledge, looking at recent national trends in the surgical timing of hip interventions in pediatric CP patients.

## Conclusions

This study aimed to determine the national incidence of surgical hip interventions in the pediatric CP population based on defined age groups. Surgical hip interventions are occurring more frequently in younger patients, potentially explained by more diligent utilization of hip surveillance programs and a better understanding of hip pathology in CP patients. While this study establishes the national trend in surgical hip interventions in CP patients, further research should be focused on determining the factors leading to these observed changes, possibly through prospective studies or retrospective studies comparing surgical trends at United States-based institutions with strict hip surveillance programs versus such institutions without strict hip surveillance programs. In addition, the effects of hip surgeries occurring at earlier ages in the CP population should be evaluated for safety and efficacy.
